# Cerebral infarction after cardiac surgery

**DOI:** 10.1002/ibra.12046

**Published:** 2022-05-25

**Authors:** Shan Wei, Yi‐Ran Cao, Da‐Xing Liu, Deng‐Shen Zhang

**Affiliations:** ^1^ Department of Cardiovascular Surgery Affiliated Hospital of Zunyi Medical University Zunyi Guizhou China

**Keywords:** brain protection, cardiac surgery, extracorporeal circulation postoperative cerebral infarction, research review, risk factors

## Abstract

Cerebral infarction, a common central nervous system complication after adult cardiac surgery, is one of the main factors leading to the poor prognosis of cardiac surgery patients besides cardiac insufficiency. However, there is currently no effective treatment for cerebral infarction. Therefore, early prevention and diagnosis of postoperative cerebral infarction are particularly important. There are many factors and mechanisms during and after cardiac surgery that play an important role in the occurrence of postoperative cerebral infarction, such as intraoperative embolism, systemic inflammatory response syndrome, atrial fibrillation, temperature regulation, blood pressure control, use of postoperative blood products, and so forth. The mechanism by which most risk factors act on the human body, leading to postoperative cerebral infarction, is not well understood, and further research is needed. Therefore, this paper aims to summarize and explain the relevant risk factors, mechanisms, clinical signs, imaging characteristics, and early diagnosis methods of cerebral infarction complications after cardiac surgery, and provides useful data for the establishment of related diagnosis and treatment standards.

## Graphical abstract

This paper mainly describes the relevant risk factors, clinical signs, imaging features, early diagnosis methods, and early preventive measures of complicated cerebral infarction after cardiac surgery.

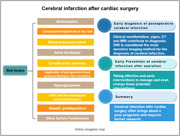

## 1 INTRODUCTION

**Figure 1:**
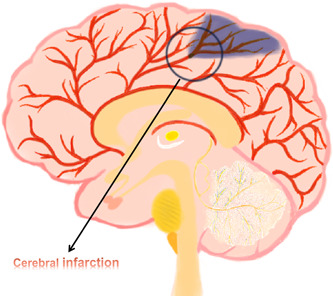
Cerebral infarction. This figure depicts the cerebral blood transport in the event of a cerebral infarction. [Color figure can be viewed at wileyonlinelibrary.com]

Cerebral infarction (Figure 1), also known as ischemic stroke and referred to as stroke in traditional Chinese medicine, is the main reason for blood supply disorder in local brain tissue areas, which can lead to hypoxia, ischemia, and necrosis of the brain tissue. As one of the severe complications that arise after cardiac surgery, it often leads to adverse outcomes in patients, not only extending the hospitalization time and increasing the financial burden on the family but also increasing the rate of disability. Data have shown that the incidence of surgical brain injury in noncardiac, nonneural, and nonlarge vessels is <1%, while it can reach 1%–3% in large vascular and cardiac surgery.^1^ Therefore, early prevention, evaluation, and treatment of cerebral infarction after cardiac surgery deserve further exploration and research.

## 2 EARLY PREVENTION AND DIAGNOSIS OF CEREBRAL INFARCTION AFTER CARDIAC SURGERY

### 2.1 Early prevention of cerebral infarction after the operation

The pathogenesis of cerebral infarction is complicated and multifaceted.  The risk factors for cerebral infarction after cardiac surgery in adults are divided into non‐modifiable factors (such as age and medical history) and modifiable factors (such as embolism, hypotension, hyperglycemia, high temperature, and surgical methods). Taking effective and early intervention measures to manage or even change these risk factors that can be intervened is an important step in reducing the incidence of postoperative cerebral infarction. Early management and intervention of related risk factors for postoperative cerebral infarction, such as prevention of intraoperative embolism, management of blood pressure and body temperature, rational application of perioperative blood products, identification and supportive treatment of previous cardiovascular and cerebrovascular diseases, and drug treatment for postoperative neuroinflammatory reactions, can reduce the incidence of cerebral infarction. After the onset of cerebral infarction, the pathogenic factors should be identified as soon as possible, and an early active intervention should be initiated to prevent stroke recurrence, which is the secondary prevention of cerebral infarction. The primary purpose is to prevent or reduce the risk of another stroke and reduce the degree of disability.

Among the risk factors for cerebral infarction after cardiac surgery, intraoperative embolization is considered to be one of the most important mechanisms. In addition, SIRS and neuroinflammation after major surgical trauma also play crucial roles in the occurrence and development of postoperative cerebral infarction. Multiple factors can cause intraoperative embolism, such as arteriovenous cannulation, left ventricular opening, cardiopulmonary bypass (CPB), improper intraoperative operation, and atherosclerotic plaque shedding. Therefore, currently, there are no effective preventive measures against the occurrence of intraoperative embolism. Also, research shows that pharmacological and nonpharmacological protective measures can be adopted to reduce the severity of postoperative systemic inflammatory response syndrome (SIRS) and neuroinflammation after major surgical trauma. Drug‐based protective measures mainly exert a protective effect on brain tissue through their inhibitory effects on inflammation. Nondrug protection measures can minimize the production of emboli mainly through the management of blood pressure, temperature management, hematocrit, and so on, thereby protecting brain tissues. Medicinal protection measures mainly include the application of anti‐inflammatory drugs (such as corticosteroids, usastatin, aprotinin, ketamine, and tetracycline derivative minocycline) and non‐anti‐inflammatory neuroprotective drugs (such as diazazine, nitric oxide, and the α2 receptor agonist dexmedetomidine). However, there is no consensus on the effectiveness of prophylactic use of anti‐inflammatory drugs for neuroprotection during the cardiac perioperative period. It has been shown that systemic inflammation can be inhibited pharmacologically, and it has been suggested that corticosteroid application during CPB may decrease the release of neuron‐specific enolase (NSE, a marker of brain damage) and the S100β protein, although this remains controversial.^2^ Similarly, Aida Salameh et al. believe that the impact of glucocorticoids on CPB‐induced inflammation remains to be discussed.^3^ There are two considerations: first, due to the complexity of the inflammatory response, even with a large number of glucocorticoids, not all glucocorticoids are used; second, although the inflammation is effectively suppressed, the clinical prognosis of patients often does not show a positive effect. Therefore, inhibiting inflammation may be a useful approach, but it remains to be demonstrated whether drug intervention can improve neurological outcomes and whether the inhibitory effect of drugs on inflammation also exerts a protective effect on neurological outcomes.^4^, ^5^, ^6^

### 2.2 Early diagnosis of postoperative cerebral infarction

Patients who show localized signs of the nervous system after cardiac surgery such as hemiplegia, aphasia, and other focal neurological dysfunctions during quiet rest, or other focal brain symptoms, generally have no obvious disturbance of consciousness, and the possibility of cerebral infarction should be considered. Brain computed tomography (CT) or brain magnetic resonance imaging (MRI) can be done in a timely fashion to help confirm the diagnosis. As an emerging neuroimaging technology, diffusion‐weighted imaging (DWI) has an extremely high display rate of acute and hyperacute cerebral infarctions and is considered to be the most sensitive imaging method for diagnosing cerebral infarction.^7^ Experimental results indicate that DWI imaging shows positivity earlier than traditional Tl and T2 imaging after brain injury. Compared with traditional MRI, DWI imaging is more sensitive. Transcranial Doppler (TCD) and near‐infrared spectroscopy (NIRS) technologies are commonly used noninvasive methods for evaluating brain perfusion. Brain electrophysiology can be a convenient and noninvasive evaluation method of brain function.

## 3 RISK FACTORS AND MECHANISMS OF CEREBRAL INFARCTION AFTER CARDIAC SURGERY

### 3.1 Embolization

**Figure 2:**
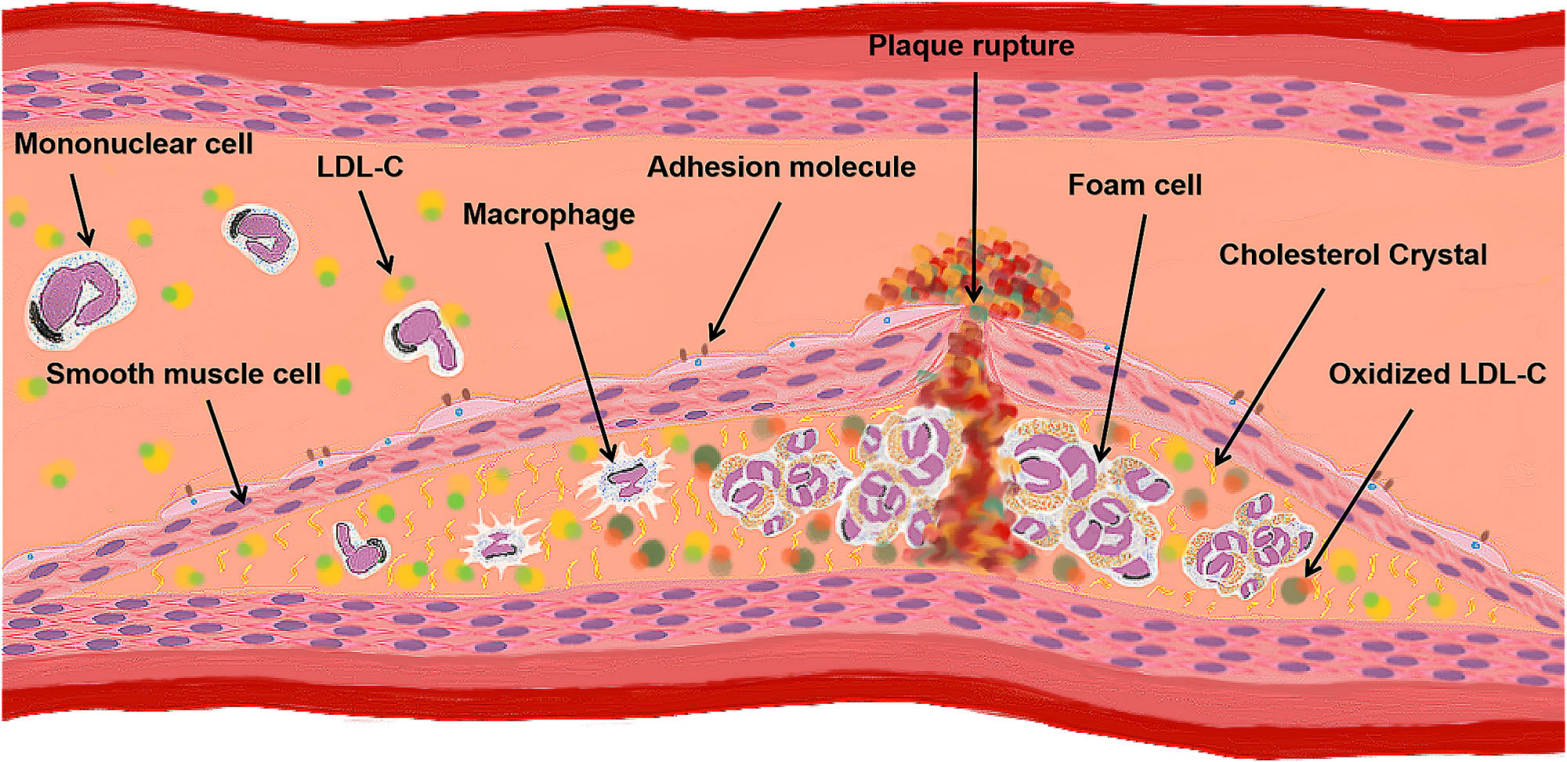
Atherosclerotic plaque rupture. This image depicts the changes that occur in blood vessels when an atherosclerotic plaque ruptures. LDL‐C, low‐density lipoprotein cholesterol. [Color figure can be viewed at wileyonlinelibrary.com]

Intraoperative embolization is considered one of the most important mechanisms of postoperative cerebral infarction, but its cause is difficult to determine or analyze. Various factors such as arteriovenous intubation, left ventricular opening, and improper intraoperative operation during in vitro circulation (cardiopulmonary bypass, CPB) may cause gas embolism.^8^ Since the solubility and gas density of carbon dioxide (CO_2_) in blood and tissue are higher than those of oxygen (O_2_), the use of CO_2_ for the whole extracorporeal pipeline before the start of extracorporeal circulation can not only decrease the formation of tiny gas embolism in the prefilled fluid but also reduce the possibility of a large amount of gas inadvertently entering into the ascending aorta,^9^ thus reducing the incidence of cerebral infarction. Some scholars believe that hyperbaric oxygen therapy (HOT) treatment can be applied to patients who have delayed awakening or neurological symptoms after CPB cardiac surgery once absolute contraindications are excluded.^8^ On the other hand, emboli made of atherosclerotic plaque fragments (Figure 2), fat, and other surgical‐derived particulate matters can cause solid embolization. At present, postoperative cerebral arterial air embolism remains an underdiagnosed, underreported disease with high morbidity and mortality. During an open‐heart surgery, the aorta is involved and its clamping and opening may lead to the shedding of the atherosclerotic plaque and eventually postoperative cerebral infarction. Therefore, some scholars believe that the degree of atherosclerosis, especially aortic atherosclerosis, is positively associated with brain injury. Studies have found that a substance called lipid microemboli (LME) is formed in the pericardial, attracting blood.^10^ It has been found in the brain of many patients who die after cardiac surgery, and it can cause dilatation of the small vascular capillaries. In addition, the lipid‐loaded embolus can also return to the arterial casing due to inadequate filtration, causing the thrombosis of the small capillaries in the brain, and ultimately leading to the occurrence of cerebral infarction.

### 3.2 SIRS and neuroinflammatory responses after major surgical trauma

SIRS and the neuroinflammatory response following cardiac surgery trauma are associated with post‐cardiac surgery brain injury.^3^, ^4^, ^11^ Blood–brain barrier (BBB) dysfunction occurs in more than 50% of patients receiving cardiac surgery.^5^ Systemic inflammatory mediators can enter the brain through a compromised BBB. The central nervous system is very sensitive to systemic inflammatory mediators such as endotoxin, cytokine interleukin‐6 (IL‐6), and IL‐8 produced during cardiac surgery.^6^

As mentioned above, intraoperative gas or solid emboli due to various reasons (such as arteriovenous cannulation, left ventricular opening, and aortic clamping) may block cerebral arteries and cause cerebral infarction. It is worth noting that in addition to cerebral ischemia–hypoxic injury, cerebral microemboli may also lead to secondary inflammatory responses. On the one hand, due to the contact between the patient's blood and the extracorporeal circulation machine during the surgery or the stimulation of various operations during the surgery, coupled with the long CPB time, a series of inflammatory reactions could be triggered. On the other hand, leukocytes, endothelial cells, and platelets in the blood are in direct contact with various inorganic and organic particles in the circulation, and these cells are activated to further release various activated proinflammatory cytokines, such as tumor necrosis factor‐α (TNF‐α), IL‐1, IL‐6, IL‐8, and prostaglandins. In addition, Toll‐like receptors (TLRs) are receptors that cannot be ignored in the inflammatory cascade. It is widely believed that TLRs are important mediators of autophagy and neuroinflammation cascades,^12^ and ligands that can bind to TLRs exist in substances released from ischemic neurons, thereby activating TLR signaling pathways and mediating inflammatory cytokines. It is released in large quantities, thereby causing secondary inflammatory damage after cerebral ischemia. This provides new ideas for the prevention and treatment of secondary inflammatory injury after severe cerebral ischemia. However, TLR‐related cerebral ischemic injury can be reduced by the establishment of TLR ischemic tolerance and TLR ligand‐induced preconditioning. In addition, some studies have found that TLR signaling pathway regulation after the occurrence of cerebral ischemia can also reduce ischemic damage and thus has a significant therapeutic effect on cerebral ischemia.^13^ Therefore, for the prevention and treatment of cerebral ischemia, TLRs should be considered as an important target.

In addition, some scholars have put forward some viewpoints that need to be further studied and verified. For example, Zhang et al. conducted a prospective study involving 88 patients undergoing cardiac surgery, and the results suggested that serum neurofilament light‐chain protein (NFL) can be used as an early and sensitive serum marker for postoperative stroke prediction in patients with acute type A aortic dissection (ATAAD), especially at 12 h after surgery.^14^ In the past, NFL has received little attention in the assessment of neurological prognosis in the field of cardiac surgery, and it is widely believed that NSE and S100β protein can be used as marker proteins to predict the neurological status of patients after cardiac surgery.^15^ However, these two marker proteins are not specific, and they are not only expressed in neurons but also found elsewhere. For example, NSE can be expressed in blood cells, while S100β can be expressed in adipose and other tissues or organs,^16^, ^17^ suggesting that these two proteins do not have specific advantages in identifying serological changes related to brain injury after cardiac surgery. NFL, the main component of the neurofilament core, is also the main intermediate filament protein of neurons and axons. Compared with NSE and S100β proteins, NFL has higher specificity in neurons, which means that serum NFL levels may be more reliable and sensitive in predicting stroke after cardiac surgery. In a study carried out by Saller et al.,^18^ it was found that NFL levels were significantly elevated in patients with postoperative psychosis after CPB. From this, the authors speculated that the increased blood NFL levels after CPB may be related to thoracotomy trauma and SIRS secondary to neuroinflammation. Systemic inflammatory mediators enter the central nervous system through the damaged BBB, eventually leading to neuronal damage. At the same time, when neurons are damaged, NFL, as an ideal brain injury biomarker protein, can pass through the BBB, so it becomes detectable in serum. Studies have shown that NFL levels in the blood continue to increase during the acute phase of stroke and peak within 3 months,^19^ and high NFL levels are also associated with poor clinical outcomes.^20^ Therefore, blood NFL levels measured in the acute phase after stroke are considered to have predictive value. However, studies have confirmed that blood NFL levels in healthy individuals are positively correlated with age, indicating that blood NFL levels reflect not only cerebral infarction‐induced secondary neurodegeneration but also age‐related neurodegeneration. After adjusting for age, sex, hypertension, and recurrent ischemic lesions,^21^ it was found that blood NFL levels were still associated with secondary neurodegeneration. In the authors' opinion, the above study shows that blood NFL levels are worthy of consideration as biomarkers of cerebral infarction‐induced secondary axonal damage, not only playing a positive prognostic role but also helping to assess the neurological effectiveness of functional recovery.

In the past, it was difficult for us to assess the neurological status of patients (mechanical ventilation, postoperative sedation, CT, MRI limitations, etc.) during and after surgery accurately and in advance. Therefore, the assessment of brain injury predictors was often performed after cardiac surgery. More prospective clinical trials are required to test whether serum NFL level is a valuable biomarker for prediction of early brain injury after cardiac surgery.

### 3.3 Atrial fibrillation (AF)

AF that occurs after surgery and during hospitalization is called postoperative AF (POAF). About half of adult patients who have undergone cardiac surgery will develop POAF, most often 2 or 3 days after surgery.^22^, ^23^ Studies have shown that the causes of POAF are diverse and may be related to inflammation, oxidative stress, and autonomic dysfunction.^24^ Patient age (>60 years), chronic obstructive pulmonary disease (COPD), left ventricular insufficiency (left ventricular ejection fraction [LVEF] < 40%), chronic kidney disease, mitral valve surgery, and discontinuation of β‐blockers have also been identified as risk factors for POAF.^25^ POAF can lead to atrial contraction, which in turn leads to a decrease in cardiac output (CO) and diastolic filling time, and thereby increases the possibility of systemic embolism, especially cerebral embolism. Cerebral embolism not only leads to prolonged hospital stay and increased hospitalization costs but also increases the risk of poor prognosis in patients. The authors believe that for high‐risk patients who may develop AF after surgery (those who have two or more relevant risk factors), appropriate interventions should be provided to reduce the incidence of postoperative AF.

The current preventive treatment of POAF mainly includes two parts: drug treatment and nondrug treatment. Adrenergic blockers or beta‐blockers (BBs) are the most widely used drugs for the prevention and treatment of postoperative supraventricular arrhythmias (including AF), and these two classes of drugs work mainly by antagonizing the effects of adrenaline. Among them, metoprolol, the most widely used drug for the prevention of POAF, is usually administered at least 48–72 h before surgery, and the dose is generally 50–200 mg. The authors suggest that BBs can be used as first‐line therapy for the prevention of postoperative AF, especially in those patients considered to be at high risk of developing POAF. In addition, discontinuation of BBs is also a related risk factor for POAF and thus should be avoided after surgery. In addition, amiodarone exerts a sympatholytic effect by blocking the α‐ and β‐adrenergic receptors, and thus can also play a role in the prevention of POAF. However, amiodarone has side effects that can lead to fatal arrhythmias such as tachycardia or ventricular fibrillation. Once these side effects occur, most patients need to reduce the dose or even stop the drug. Therefore, amiodarone is not considered a first‐line drug for the prevention of POAF, and it is mainly considered in patients with contraindications to the use of BBs. However, it is worth noting that the above drugs also have their own side effects. BBs may cause hypotension and atrioventricular block. For example, the class III antiarrhythmic drug amiodarone may cause tachycardia or ventricular fibrillation and other fatal arrhythmias. Therefore, from the authors' point of view, the impact of the side effects of the drug itself on patients should be minimized through alternate and rational drug regimens.

For nondrug preventive measures, Mulay et al. developed the posterior pericardiotomy (PP) technique.^26^ At the end of the procedure, a longitudinal incision is made parallel to the posterior of the phrenic nerve, extending from the left inferior pulmonary vein to the diaphragm. Two pleural drains are inserted, one in the left pleural space and the other in the anterior mediastinum, to adequately drain the pericardial effusion, thereby reducing POAF. Xiong et al. conducted a systematic review and meta‐analysis consisting of 10 randomized‐controlled trials to investigate the feasibility of PP in the prevention of new‐onset AF after coronary artery bypass grafting.^24^ They considered PP to be a simple and safe surgical approach with nonobvious complications. However, the number of randomized‐controlled trials that they included is small, the quality of the relevant research data is low, and thus the conclusion of the research is weakened. More high‐quality randomized‐controlled trials are still needed to further evaluate the feasibility and safety of PP in preventing POAF. The authors believe that, compared with the unavoidable side effects caused by drug treatment, nondrug preventive measures are simple, effective, and economical, and can effectively shorten the length of hospital stay, save medical and material resources, reduce the occurrence of adverse events, and improve patient outcomes. However, as mentioned above, more research and experiments are still needed for its verification.

### 3.4 Temperature control during CPB

It is well known that the brain metabolic rate is directly related to temperature, and the cerebral oxygen metabolic rate is often reduced by low temperature in cardiac surgery to reduce cerebral hypoxia and brain damage during CPB. Conversely, increased brain temperature can aggravate ischemia and accelerate nerve cell death. In addition, elevated brain temperature can also delay the recovery of neural cell metabolism and the increased release of excitotoxic neurotransmitters.^27^ However, what we cannot ignore is that hypothermia has its own adverse effects; for example, it hinders the uptake of oxygen and glucose in the patient's brain and causes brain cell edema, which further leads to transient intracranial pressure increase and long‐term neurophysiological dysfunction.

An earlier study showed that patients receiving the normothermic CPB (i.e., >35°C) had three times the risk of stroke compared with patients receiving the hypothermic CPB (i.e., <28°C).^28^ However, despite numerous studies, the exact scope of temperature management during cardiac surgery remains controversial.^29^ Current clinical recommendations focus on avoiding hyperthermia (blood temperature at arterial outlet ≥37°C) during surgery, and once the temperature exceeds a certain range (30°C), the rewarming rate is reduced accordingly (≤0.5°C/min).^30^

In recent years, the normothermic CPB (NCPB) technique has been gaining popularity. Clinicians are also becoming more adept at applying the technique in adult cardiac surgery. For children with congenital heart disease, hypothermic cardiopulmonary bypass (HCPB) is usually adopted.^31^ In HCPB, CPB is used only for cooling and rewarming, and it simplifies venous cannulation so as to provide the surgeon with adequate intraoperative exposure. In recent years, some scholars have found that compared with HCPB, NCPB may have more advantages in reducing oxidative stress in cardiomyocytes.^32^ In addition, NCPB avoids the cooling and rewarming time required at the beginning and end of HCPB, thereby shortening the time required for CPB. NCPB also reduces the time spent on inotropes and respiratory support, while avoiding the reduced blood flow and hemodilution required in HCPB. This not only shortens hospital stays but also reduces perioperative blood transfusion requirements. It is undeniable that there are also some potential problems with the use of NCPB, such as insufficient surgical exposure and inadequate neuroprotection. In the authors' opinion, its clinical advantages greatly outweigh its potential risks, and the advantages outweigh the disadvantages. However, there is no strong evidence to date that NCPB is superior to HCPB in reducing adverse events and mortality after cardiac surgery.^31^ More prospective multicenter randomized‐controlled trials are required to explore and confirm the advantages of NCPB in children with congenital heart disease.

### 3.5 Blood pressure control

The goal of optimal blood pressure control during CPB in cardiac surgery has always been the center of debate.^33^, ^34^ During extracorporeal circulation, when the average arterial pressure is less than 64 mmHg, the mean arterial pressure is closely correlated with stroke onset.^26^ When patients with cardiac surgery develop postoperative hypotension, the blood flow rate slows down, which not only reduces the removal of emboli but also increases blood viscosity. Increased blood viscosity may lead to hypoperfusion of the cerebral blood vessels, which easily induces thrombosis. Therefore, insufficient cerebral blood perfusion can not only lead to brain damage but also cerebral embolism and therefore aggravated brain injury. It is well known that the brain has a mechanism to maintain stable blood flow in the face of fluctuating blood pressure, which is called cerebral blood flow autoregulation (standard cerebral blood flow autoregulation range 60–160 mmHg). At the same time, it has been shown that during CPB, if the increase of the mean arterial pressure exceeds the range of cerebral autoregulation, it may lead to a higher postoperative risk of postoperative delirium. Therefore, the optimal control range of blood pressure in cardiac surgery still needs to be further investigated and studied.

### 3.6 Carotid artery stenosis

Carotid atherosclerosis is an independent risk factor for stroke after cardiac surgery.^35^ Carotid artery stenosis caused by carotid atherosclerosis can lead to insufficient cerebral blood flow, which may be the main cause of brain injury, or it may be the cause of secondary cerebral embolism and further aggravates the injury. Irrespective of whether carotid atherosclerotic stenosis is treated before cardiac surgery or as part of a “combined” operation,  the incidence of postoperative stroke can be reduced. In the authors' opinion, when performing routine cardiac color ultrasound examination before surgery, it is recommended to conduct a carotid artery color ultrasound examination for high‐risk groups (hyperlipidemia, hypertension, obesity, smoking, etc.), and for early diagnosis and intervention for possible carotid artery stenosis and sclerosis.

### 3.7 Application of blood products during the perioperative period

During CPB, induced hypothermia will increase blood viscosity, so blood dilution is often used to reduce blood viscosity and decrease the need for allogeneic blood transfusion. Studies have shown that excessive blood dilution will increase the incidence of postoperative cerebral infarction.^36^ In clinical practice, it is not uncommon to inject white floating red blood cells to correct patient anemia and improve blood oxygen‐carrying capacity. Studies have shown that patient age, preoperative creatinine clearance, hemodialysis, total body surface area, preoperative hemoglobin (Hb), history of thoracic and cardiac surgery interventions, and preoperative cardiogenic shock are significantly associated with red blood cell transfusions after cardiac surgery.^37^ In addition, red blood cell transfusions of more than 2 U after cardiac surgery increase the risk of stroke or transient ischemic attack by three or four times.^38^ The underlying mechanisms may be related to impaired oxygen transport at the cellular level, prothrombotic events secondary to abnormal red blood cell morphology, and the release of harmful substances from erythrocytes.^39^, ^40^ Barhainwala et al. believe that the postoperative Hb levels and the amount of suspended red blood cells infused during the operation are independent influencing factors of postoperative cerebral infarction.^41^ However, the study by Chen Fei et al. did not find a direct relationship between cerebral infarction after coronary artery bypass grafting and the infusion of suspended red blood cells.^41^ This is contrary to the results of Mariscalco et al.,^38^ and is somewhat controversial.

### 3.8 Hyperglycemia

The neurocognitive function is also affected by the level of serum glucose.^42^ Even a slight increase in blood sugar (>7.8 mmol/L [140 mg/dl]) can affect the prognosis of stroke patients through various mechanisms.^2^ Among nondiabetic patients, a postoperative target blood glucose control range of 4.4–6.1 mmol/L can significantly reduce the incidence of neurological adverse events.^43^ On the one hand, due to the stress response after cardiac surgery, the sensitivity of peripheral insulin can be reduced, thereby causing blood glucose to increase; on the other hand, many patients undergoing cardiac surgery have diabetes before surgery, and exposure to high glucose syndrome can cause physiological compensatory reactions such as downregulation of glucose transporters in brain capillaries, thus reducing the amount of glucose entering the brain. This response also explains why intraoperative hyperglycemia may be more devastating during brain damage in nondiabetic patients than in diabetic patients.

### 3.9 Genetic predisposition

Studies have suggested that genetic susceptibility can contribute to individual susceptibility and variability in postcardiac brain injury. Apolipoprotein 84 gene and the platelet antigen‐II (PLA‐II) receptor has been receiving attention. Apolipoprotein 84 may increase the risk of cognitive impairment, which may be related to the occurrence of atherosclerosis. PLA‐II plays an important role in acute coronary syndrome and thrombosis.^44^ However, the etiological mechanism of the above genes responsible for causing cerebral infarction after cardiac surgery is currently unknown, and further research is needed.

## 4 SUMMARY

Brain infarction, one of the most common complications after cardiac surgery, has received increasing attention in recent years because of its serious consequences such as poor prognosis and even death. The risk factors for postoperative cerebral infarction can be divided into nonmodifiable parts (e.g., age and previous medical history) and modifiable parts (e.g., embolism, hypotension, hyperglycemia, high body temperature, and surgical methods). The focus of brain protection is on intervening with modifiable risk factors that can reduce the occurrence of postoperative cerebral infarction. The clinical characteristics of postoperative cerebral infarction are primarily manifested in the fact that CPB surgery is more likely to cause bilateral cerebral hypoperfusion, mostly bilateral and large‐area cerebral infarction, and the clinical symptoms of patients are more severe, but cerebral infarction after nonpump surgery is mostly cortical infarction. In terms of early diagnosis, DWI is more sensitive than traditional MRI and is considered the most sensitive imaging method for diagnosing cerebral infarction. The protective measures for brain tissue are divided into drug protective measures and nondrug protective measures, but whether the current anti‐inflammatory drugs can effectively enhance the postoperative SIRS and the neuroinflammatory response after major surgical trauma remains controversial. In general, the current research on the prevention of cerebral infarction after cardiac surgery is far from perfect, and strong evidence‐based medical evidence is needed to provide further guidance for future clinical diagnosis and treatment.

## AUTHOR CONTRIBUTIONS

Shan Wei researched and organized the literature, designed the paper framework, drafted the paper, and revised the paper. Yi‐Ran Cao helped to collect and collate the data. Da‐Xing Liu and Deng‐Shen Zhang provided research funding, technical or material support, and guidance.

## TRANSPARENCY STATEMENT

All the information in this article is open and transparent.

## CONFLICTS OF INTEREST

The author declares no conflicts of interest.

## ETHICAL STATEMENT

Not applicable.

## Data Availability

Data sharing is not applicable to this article as no new data were created or analyzed in this study.
